# Evaluation of a carepartner-integrated telehealth gait rehabilitation program for persons with stroke: study protocol for a feasibility study

**DOI:** 10.1186/s40814-023-01411-1

**Published:** 2023-11-24

**Authors:** Sarah Blanton, George Cotsonis, Kayla Brennan, Robert Song, Laura Zajac-Cox, Sarah Caston, Heather Stewart, Arun Jayaraman, Darcy Reisman, Patricia C. Clark, Trisha Kesar

**Affiliations:** 1 Division of Physical Therapy, Department of Rehabilitation Medicine, Emory University School of Medicine, 1441 Clifton Road NE, Room 213, Atlanta, GA 30322 USA; 2 Department of Biostatistics and Bioinformatics, Emory University Rollins School of Public Health, 1518 Clifton Road, NE, Atlanta, GA 30322 USA; 3 Emory Rehabilitation Hospital, Atlanta, GA USA; 4 Technology & Innovation Hub (tiHUB), Department of Physical Medicine and Rehabilitation, Department of Physical Therapy and Human Movement Sciences, Feinberg School of Medicine, Max Näder Center for Rehabilitation Technologies & Outcomes Research, Northwestern University, Chicago, IL 60611 USA; 5 Department of Physical Therapy and Graduate Program in Biomechanics and Movement Science, Neurologic and Older Adult Clinic, University of Delaware, Newark, DE USA; 6 Byrdine F. Lewis School of Nursing, Georgia State University, Atlanta, GA USA

**Keywords:** Caregiver, Stroke, Rehabilitation, Telehealth, Gait, Dyads

## Abstract

**Background:**

Despite family carepartners of individuals post-stroke experiencing high levels of strain and reduced quality of life, stroke rehabilitation interventions rarely address carepartner well-being or offer training to support their engagement in therapeutic activities. Our group has developed creative intervention approaches to support families during stroke recovery, thereby improving physical and psychosocial outcomes for both carepartners and stroke survivors. The purpose of this study is to test the feasibility of an adapted, home-based intervention (Carepartner Collaborative Integrative Therapy for Gait-CARE-CITE-Gait) designed to facilitate positive carepartner involvement during home-based training targeting gait and mobility.

**Methods:**

This two-phased design will determine the feasibility of CARE-CITE-Gait, a novel intervention that leverages principles from our previous carepartner-focused upper extremity intervention. During the 4-week CARE-CITE-Gait intervention, carepartners review online video-based modules designed to illustrate strategies for an autonomy-supportive environment during functional mobility task practice, and the study team completes two 2-h home visits for dyad collaborative goal setting. In phase I, content validity, usability, and acceptability of the CARE-CITE-Gait modules will be evaluated by stroke rehabilitation content experts and carepartners. In phase II, feasibility (based on measures of recruitment, retention, intervention adherence, and safety) will be measured. Preliminary effects of the CARE-CITE-Gait will be gathered using a single-group, quasi-experimental design with repeated measures (two baseline visits 1 week apart, posttest, and 1-month follow-up) with 15 carepartner and stroke survivor dyads. Outcome data collectors will be blinded. Outcomes include psychosocial variables (family conflict surrounding stroke recovery, strain, autonomy support, and quality of life) collected from carepartners and measures of functional mobility, gait speed, stepping activity, and health-related quality of life collected from stroke survivors.

**Discussion:**

The findings of the feasibility testing and preliminary data on the effects of CARE-CITE-Gait will provide justification and information to guide a future definitive randomized clinical trial. The knowledge gained from this study will enhance our understanding of and aid the development of rehabilitation approaches that address both carepartner and stroke survivor needs during the stroke recovery process.

**Trial registration:**

ClinicalTrials.gov, NCT 05257928. Registered 25 February 2022.

**Trial status:**

This trial was registered on ClinicalTrials.gov (NCT 05257928) on March 25, 2022. Recruitment of participants was initiated on May 18, 2022.

## Background

Recognized as an independent risk factor for other comorbidities and a recurrent stroke [1], reduced physical activity in stroke survivors is a critical area of rehabilitation focus [2]. Few stroke survivors meet the current American Stroke Association guidelines [3] of 20–60 min of aerobic exercise 3 to 5 days per week. Typically, stroke survivors achieve only 63% of the recommended steps per day for people with disability (4078 versus 6500–8500) [4]. According to Fini et al. [2], motivation and carepartner support are potential factors to consider, underscoring self-management and behavior change to enhance engagement and sustainability of poststroke physical activity programs. However, lacking training and preparation to support the complexities of stroke rehabilitation [5–7], carepartners can experience high levels of care burden, reduced quality of life, and family conflict surrounding the poststroke recovery process [8, 9]. Designing interventions that support physical activity by engaging family carepartners may be a key factor for the sustainability of health-related behaviors.

Carepartner’s well-being can impact the health outcomes of both the carepartner and stroke survivor, as the physical, emotional, and psychological aspects of their daily living are intricately interconnected. With earlier discharge and a greater proportion of rehabilitation occurring at home, it is critical to find strategies to enable carepartners to support rehabilitation at home and in the community without adding to their own caregiving burden. Previous research studies incorporating carepartner training during stroke survivor rehabilitation have had promising results [10, 11]. The London Stroke Carers Training Course, a comprehensive competency training for carepartners, reduced health and social care costs, improved QOL, and reduced carepartner burden [11]. However, these findings were not replicated in a multi-site study [12], which may reflect the limitations of approaches that prescribe activities for carepartners instead of actively involving them as collaborators in the rehabilitation process. Work by Creasy et al. [13] suggests that carepartners are keenly aware of the critical nature of their role in the care of their loved one, and that they have expectations of being included in poststroke treatment planning. Carepartners underscored their need to have greater information about stroke and customization of rehabilitation training to more accurately address specific family needs [14]. Several studies have shown that specifically improving carepartner coping and life skills to care for a chronically ill family member leads to decreased caregiver burden and improved QoL [15–17]. Supporting previous recommendations [7, 17], a recent systematic review of stroke family carepartner and dyad interventions [18] emphasized that these interventions should combine skill building (e.g., problem-solving, goal setting, and stress management) with psychoeducational strategies and should be tailored to individualized needs of the carepartner. Collectively, these studies suggest that the engagement of carepartners offers valuable opportunities to enhance rehabilitation therapies.

Indeed, the delivery of interventions without consideration of family context may limit the success of stroke recovery. To address this critical gap, we developed a theory-based, task-specific, and carepartner-focused intervention — Carepartner and Collaborative Integrated Therapy (*CARE-CITE*) [19]. Initially CARE-CITE was designed to enhance upper extremity functional task practice (repetitive performance of daily activities in the home setting to improve function) by instructing carepartners in methods for collaborative goal setting, problem-solving, and creating an autonomy-supportive environment. Arising from self-determination theory [20], CARE-CITE uses web-based interactive modules with exemplary videos created in collaboration with actual stroke dyads (carepartners and stroke survivors), which model autonomy supportive communication by offering choice, providing rationale, demonstrating empathy, and avoiding controlling language. Preliminary studies show the feasibility [21] and promising therapeutic benefits of CARE-CITE coupled with upper extremity constraint-induced movement therapy (CIMT). In our baseline data with chronic stroke survivors [22], we found that the majority of carepartners continue to experience family conflict surrounding stroke recovery which was related to higher levels of carepartner strain and less autonomy support provided to the stroke survivor during rehabilitation activities. These findings provide insights into the potential influence of family context on stroke survivor motivation and adherence. Outcomes from our study showed promising trends suggesting that carepartners receiving CARE-CITE had improved psychosocial outcomes, including improved quality of life, coupled with less strain, fatigue, and family conflict around stroke recovery [23, 24]. Although both stroke survivor control (CIMT-only) and intervention (CARE-CITE + CIMT) groups demonstrated improvements in upper extremity function and health-related quality of life [24], only the CARE-CITE + CIMT group maintained or continued to improve upper extremity function at 1-month follow-up testing. These results suggest that carepartner engagement may be instrumental for continued progress during stroke recovery.

Responding to the insights gained from the engagement of the carepartner in upper extremity rehabilitation, we now seek to broaden the scope of our intervention and further optimize poststroke recovery by pairing CARE-CITE with home-based gait and functional mobility training (CARE-CITE-Gait). The aim of this paper is to describe the CARE-CITE-Gait, two-phased, feasibility study design. The objective of Phase I of this trial is to evaluate the content validity and user satisfaction (usability and acceptability) of the CARE-CITE-Gait intervention. The primary objective of phase II is to determine the feasibility of CARE-CITE-Gait for stroke survivor and carepartner dyads, as indicated by participant recruitment and retention, adherence to the intervention, and safety (occurrence of stroke survivor or carepartner adverse events). The secondary objective of Phase II is to determine the preliminary effects of the CARE-CITE-Gait intervention on stroke survivors and carepartners. Carepartner psychosocial outcomes will include family conflict about stroke recovery, strain, autonomy support, and quality of life. Stroke survivor outcome measures include functional mobility, gait speed, stepping activity, and health-related quality of life. Insights from this study will guide the development of a randomized clinical trial to evaluate the efficacy of the CARE-CITE-Gait intervention.

## Methods

Identification and reporting of relevant elements of this protocol are based on the Standard Protocol Items: Recommendations for Intervention Trials (SPIRIT) checklist [25] and Template for Intervention Description and Replication (TIDieR) guidelines for intervention descriptions [26]. This is the first published version of this clinical trial study protocol. Ethical approval was obtained by Emory University Institutional Review Board, and this protocol is registered on ClinicalTrials.gov (NCT05257928). Any protocol amendments will be immediately reported to the University Institutional Review Board for approval and to the funding agency as appropriate.

### Study design and setting

**Fig. 1:**
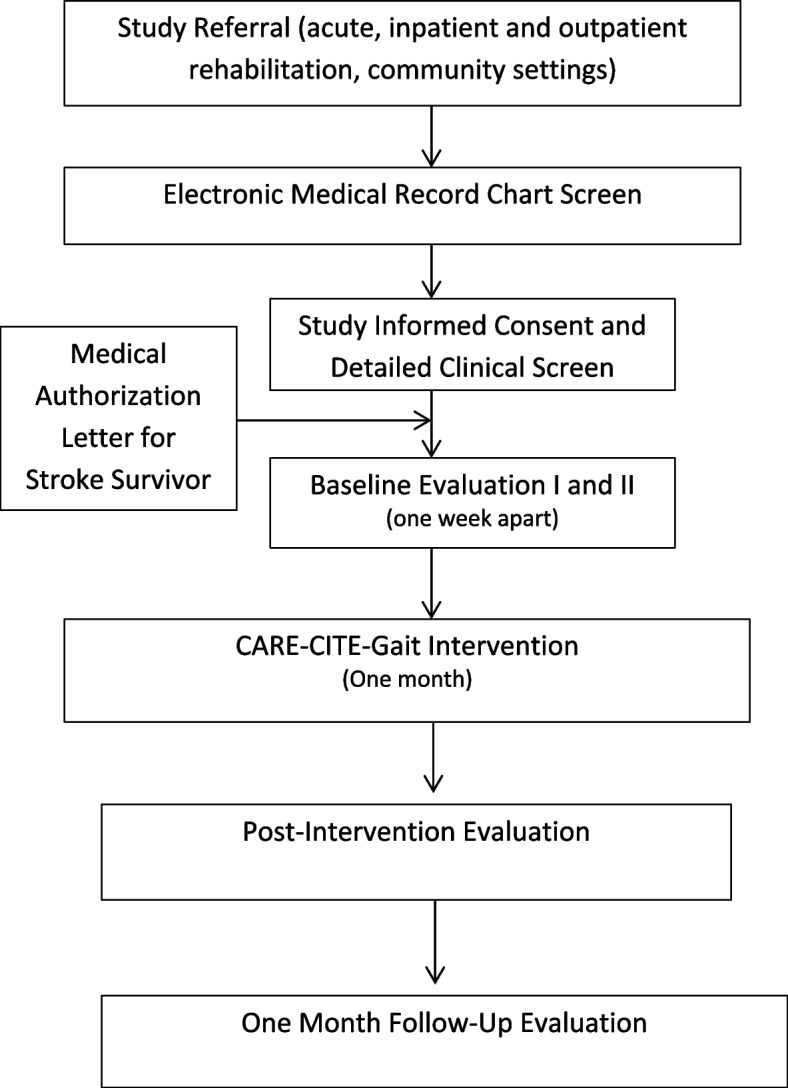
Study flow chart: Evaluation of a Carepartner-Integrated Telehealth Gait Rehabilitation Program for Persons with Stroke (CARE-CITE-Gait) (*N* = 15)

In Phase I, stroke rehabilitation content experts (physical and occupational therapists) and carepartners will evaluate the content validity and user satisfaction (usability and acceptability) of the CARE-CITE-Gait modules. In Phase II, feasibility (based on measures of recruitment, retention, intervention adherence, and safety) and preliminary effects of CARE-CITE-Gait will be measured using a one-group, quasi-experimental design with repeated measures (two baseline visits 1 week apart, posttest, and 1-month follow-up) with 15 stroke survivor and carepartner dyads. While the in-person portion of the intervention will take place in participant homes, the study recruitment, screening, and evaluations (evaluator blinded) for dyads will occur in a stroke research laboratory within an urban rehabilitation hospital located in Atlanta, GA, USA (Fig. 1 Study flow chart: evaluation of Carepartner-Integrated Telehealth Gait Rehabilitation Program for Persons with Stroke (CARE-CITE-Gait) *N* = 15)). Licensed physical therapists will conduct the evaluations and administer the 4-week home-based CARE-CITE-Gait intervention. During the intervention period, carepartners will access the web-based CARE-CITE-Gait modules independent of study staff involvement. The study schedule schematic design is presented in Table 1.

**Table 1:**
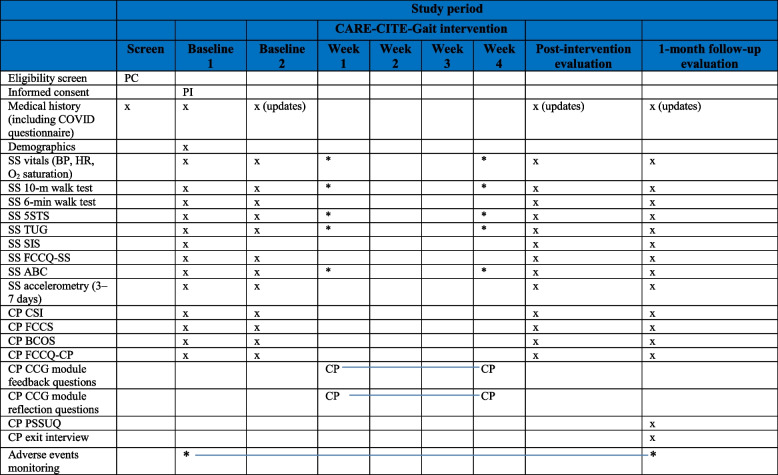
CARE-CITE-Gait study schedule

*SS* stroke survivor, *CP* carepartner, CCG-CARE-CITE-Gait, *BP* blood pressure, *HR* heart rate, *O*_*2*_ oxygen saturation, *5STS* 5-times sit to stand, *TUG* Timed Up and Go test, *SIS* Stroke Impact Scale, *FCCQ-SS* Family Care Climate Questionnaire for stroke survivor, *ABC* Activities-specific Balance Confidence Scale, *CSI* Caregiver Strain Index, *BCOS* Bakas Caregiving Outcomes Scale, *FCCS* Family Caregiver Conflict around Stroke Recovery Scale, *FCCQ-CP* Family Care Climate Questionnaire for carepartner, *PSSUQ* Post-Study System Usability Questionnaire, *PC* completed by project coordinator, *PI* completed by PI, *x* completed by evaluator, *completed by intervention therapist

### Phase I: Content validity and user satisfaction of CARE-CITE-Gait

The original CARE-CITE modules were designed to help the carepartner create a therapeutic home environment while encouraging the stroke survivor to use the weaker upper extremity during daily activities. We modified the existing upper extremity CARE-CITE intervention to address gait rehabilitation. Collaborating with Emory University’s Center for Digital Scholarship, the video modules were redesigned with a progressive therapeutic exercise plan to improve overall mobility and stepping-related physical activity. Additional content was added to address safety risks and falls (appropriate use of gait belts, supervision, etc.) associated with gait and balance exercises. The gait revisions maintained the core CARE-CITE theoretical framework (the concept of autonomy support), with text and video that demonstrate ways to encourage empathy (video examples of discussions of carepartners with stroke survivors acknowledging the difficulty of the exercise), collaborating on problem-solving (examples of methods to increase or decrease the difficulty of activities together), emphasizing the importance of offering the stroke survivor choice in activities to practice (examples of joint goal setting), and ways to provide noncontrolling language (scenarios showing controlling vs. noncontrolling language).

In Phase I, we will expand upon the established validity and satisfaction demonstrated for the original upper extremity CARE-CITE intervention [21] using similar procedures to evaluate the adaptation of the modules to gait and mobility examples. To determine content validity, six to seven stroke rehabilitation content experts will review modules for content accuracy, problem relevance, and ease of use with forms adapted based on work by Bakas and colleagues [27]. Qualitative feedback with general comments and suggestions for improvement will be gathered from open-ended questions.

To assess user satisfaction (usability, acceptability, and overall satisfaction), three carepartners will complete questions at the end of each module rating the following areas: (1) usefulness of overall content, (2) usefulness of written text, (3) usefulness of videos, (4) ease of use, and (5) acceptability using a 5-point Likert-type response scale. Time required for module review and general comments and improvement suggestions will be collected. In our previous work [21], carepartners required approximately 5–20 min to complete each of the six modules. Data gathered about content validity and user satisfaction will guide any additional refinements of intervention before the initiation of Phase II study recruitment**.**

### Phase II: Feasibility and preliminary effects of CARE-CITE-Gait

For Phase II, the study coordinator will make a clinic appointment at Emory Rehabilitation Hospital for interested participants. If screening criteria are met, written informed consent will be obtained from the stroke survivor and carepartner by the study PI or study coordinator. Medical clearance from the stroke survivor physician will be obtained before study participation.

### Recruitment

#### Participants

##### Inclusion criteria

All participants must be greater than 21 years of age, able to read and write English, and able to provide informed consent. Stroke survivors will be > 3-month postischemic or hemorrhagic event and discharged home, able to walk 10 m with or without an assistive device, have no severe cognitive deficits (mini-mental test > 24 [28]) or physician-determined major medical problems that would limit participation, and have the presence of a carepartner. Carepartners will be self-identified as a spouse/partner or family member dwelling in the same household who has the role as the primary caregiver of the stroke survivor. The primary carepartner inclusion criteria include interest and willingness to support the stroke survivor during the study activities, ability to provide any necessary supervision with safety-related mobility activities, and no significant cognitive deficits (as demonstrated by their ability to explain the study purpose to the PI during the informed consent discussion). Participants will be requested not to participate in other research studies during the study intervention and evaluation period.

#### Recruitment and retention strategies

Through weekly monitoring of the inpatient and outpatient rehabilitation stroke census, potential participants will be identified by study staff, and eligibility criteria will be confirmed based on physician and physical therapy electronic medical records. To broaden recruitment outreach, stroke information in services will be provided to regional hospitals and community support groups as well as partnering with other regional stroke research collaborators to facilitate shared recruitment efforts. Targeted enrollment rate is two to three dyads per month. To support participant adherence and retention, the study coordinator and PI will be available to respond promptly to any participant concern or issue (via email or phone) and will provide regular check-in telephone calls or texts for appointment confirmation reminders. Each participant receives US $50 (US $100/dyad) for study participation.

#### Sample size estimation

For Phase II, given the time required to adapt the intervention, the proposal timeline, and preliminary data from our upper extremity intervention, a sample size of 15 dyads is proposed to evaluate feasibility. An attrition rate of ~ 8% is projected based on the literature and our previous work [29]; thus, we will enroll 16 dyads to achieve a final sample of 15. Assuming a two-tailed alpha = 0.05, we will have 80% power to detect an effect size = 0.78 (Cohen, large) and can calculate 95% confidence intervals within 0.64 standard deviations. These data will provide precision to the estimates of mean changes, variability, and effect sizes for key outcomes. Importantly, these data will inform sample size estimation and identify feasibility lessons to guide our planned next step—a randomized clinical trial evaluating the efficacy of CARE-CITE-Gait.

### Intervention

#### CARE-CITE-Gait intervention

The CARE-CITE-Gait intervention will occur over 4 weeks in the dyad’s home. In-person visits by the interventionist will occur at weeks 1 and 4, consisting of facilitation of collaborative goal setting, development of a personalized home exercise program based on daily activities related to gait and mobility, and assessment of gait speed and balance. Telephone check-ins occurring in weeks 2 and 3 will consist of a 10-min phone call with the carepartner to address any questions and identify challenges and opportunities with the implementation of autonomy-supportive strategies and problem-solving with the stroke survivor. Carepartners will complete 6 online CARE-CITE Gait modules throughout the course of the intervention using a web platform. Modules will consist of demonstration videos and instructive content covering the following topics: collaborative goal setting that addresses both stroke survivor and carepartner goals, principles of functional task practice (practice of daily activities), and strategies for task adaptation and progression to drive neuroplasticity (see Table 2). A central theme of the modules is helping carepartners to provide autonomy support for the stroke survivor by fostering empathy, incorporating choice in activities, and providing instruction in the use of noncontrolling language. An additional module encourages carepartners to create their own self-care goals.

**Table 2:** Content of the CARE-CITE-Gait intervention for the carepartner

Modules	Content
Overview of modules structure	Each module has multimedia to provide the purpose, educational content, and/or illustrate examples of the topics discussed. Concluding each module, four to five reflection questions are provided to allow for application of content, and seven feedback questions are provided to gather information on ease of use, acceptability, and usefulness of modules
Module I: Introduction to CARE-CITE-Gait	Describes CARE-CITE Gait project and defines the roles of the carepartner and stroke survivor and summarizes the modules. Welcome survey provided for the carepartner to complete with research interventionist for practice using the website and answering questionnaires
Module II: Introduction to carepartner and collaborative integrated therapy—CARE-CITE	Overview of goal setting (providing examples in the areas of household activities of daily living, leisure, and work-related and collaborative activities), home diary (to record activities and difficulty level), and review of safety measures and behavior contract (use of gait belt, agreement between stroke survivor and carepartner on individual and collaborative activities) Two videos showing examples of practicing activities of daily living to improve balance and gait, image examples of a completed home diary, and behavior contract and seven videos of conversations around safety and considerations
Module III: Practice and goal setting	Review of role of practice in promoting neuroplasticity and recovery after stroke. Discussion of collaborative problem-solving to accomplish tasks, maintaining appropriate challenge threshold, both reducing task complexity when a task is too difficult and increasing challenge when the task is easily mastered. Two to seven video clips capture each of the six themes of practice
Module IV: Autonomy support—creating partnerships	Cultivating an autonomy supportive environment with empathy, problem-solving through tasks in the home setting, use of noncontrolling language, and offering choice. Recognize challenges and explore ways to improve communication (avoid controlling language such as “you should exercise,” or “you have to do this”). Eight video clips illustrate understanding another’s viewpoint, using problem-solving strategies, providing rationale, and providing choice during gait and mobility exercises
Module V: Taking care of yourself as a carepartner	Carepartner self-care recognizing demands of caregiving role, strategies for stress reduction, opportunities for self-care activities, and community resources (only text)
Module VI: Reflections	Six videos (limited text) of stroke survivors and carepartner reflecting on rehabilitation and recovery. Encouraging carepartner reflection on his/her role in recovery of the stroke survivor

#### Standardization

To increase rigor and minimize bias, study staff collecting data will be blinded to study intervention and trained in outcome measure administration with regular assessments of competence every 1.5 months by the PI. Variability and risk for interrater reliability concerns will be minimized by using the same study staff for all assessments of a dyad when possible. The PI will provide supervision of in-home visits and phone calls for the first five dyads and will communicate through email or phone with the interventionist after each visit for each subsequent dyad to ensure consistency of intervention delivery. Evaluation and intervention data collection forms will be standardized to facilitate protocol adherence. In addition to adherence with good laboratory practice [30] principles for clinical research, all study personnel will receive ongoing university diversity, equity, and inclusion training to foster education, self-awareness, communication skills, and community engagement.

### Outcomes

#### Feasibility

Feasibility of the study protocol will be assessed by participant recruitment, retention, intervention adherence, and safety as defined in Table 3. Justification of ineligibility, declining to participate, or study withdrawal following enrollment and obtaining signed consent will be recorded in the study flowchart.

**Table 3:** CARE-CITE-Gait study feasibility outcomes

Outcome	Definition
Recruitment	Recruitment rates will be the percentage of those participants enrolled and randomized from those screened. Recruitment will be deemed feasible if the target enrollment of 15 dyads (2–3 dyads per month) is reached during the study timeframe
Retention	Retention of participants will be the percentage of dropouts. An acceptable retention rate will be 85% of enrolled participants for completion of post-evaluation (80% for 1-month follow-up)
Intervention adherence^a^	Carepartner adherence will be the number of modules reviewed (six total modules) as indicated by the reflection questions completed at the end of each section. Criteria for carepartner adherence will be a minimum completion of five of the six modules. *Study staff are electronically notified of module completion in real time which allows for reminders to be sent to participants to review modules as needed* Criteria for carepartner and stroke survivor adherence will be > 3.5 h (for two home visits) and CP completion of weeks 2 and 3 phone calls
Safety	Number and type of adverse events (serious vs. non-serious; related or possibly related to CARE-CITE-Gait)

^a^Adherence to the intervention will be evaluated only in the participants completing a baseline and a post-intervention evaluation

In addition to the above feasibility measures, at the end of the 1-month follow-up evaluation, carepartners will complete the standardized Post-Study System Usability Questionnaire [31] and a study exit interview questionnaire. This exit interview questionnaire was developed by the PI based on post-study participation interviews in similar stroke research [32] and was reviewed by stroke caregiving experts for content validity. The interview evaluates the carepartner’s perceptions of their confidence in providing care, value of participation in the study, and helpfulness of the intervention with a mix of rating scales and open-ended questions.

#### Outcomes for carepartner and stroke survivor

Carepartner psychosocial outcome measures and stroke survivor physical function and health-related quality of life outcome measures will be administered in separate rooms at the stroke research laboratory before and immediately after the 4-week intervention and at 1-month follow-up. After each assessment visit, stroke survivors will wear ankle accelerometers to measure home and community stepping activity over 7 days. Each outcome measure has been tested in the stroke population previously (Table 4 lists outcome measure descriptions with established reliability and validity).

**Table 4:** Outcome measures collected at baseline, post-intervention, and 1-month follow-up

Variable	Measure	Description	Reliability/validity
SS gait speed^a^	10-m walk test	Time to complete a standardized overground distance; correlated with functional ambulation categories	Excellent test–retest (*ICC* > 0.95) [37], intra- and inter-rater reliability (*ICCs* > 0.87); established construct [38] and criterion [39] validity in stroke
SS endurance	6-min walk test	Distance walked in 6-min; measure of aerobic capacity and long-distance walking function	SEM, MDC, and MCID published in older adults and stroke; excellent reliability (*ICC* > 0.99); validity established in stroke [38, 40, 41]
SS dynamic balance and functional mobility^a^	5-times sit to stand (5STS) Timed up and go test (TUG)	Timed tests of functionally relevant lower limb activities (sit to stand, walking, turning)	5STS—criterion validity with leg muscle strength in stroke; discriminates stroke versus able-bodied [42–44] TUG—SEM, MDC, reliability, validity published in stroke [38, 45, 46]
SS quality of life	Stroke Impact Scale (SIS)	Fifty-nine items, eight domains function	Test–retest reliability *ICC* = 0.70 to 0.92; internal consistency alpha coefficient of 0.83–0.90 [47]
SS mobility confidence^a^	Activities-specific Balance Confidence Scale (ABC)	Sixteen items, self-report of balance confidence with balance-related activities	Adequate to excellent test–retest reliability for total score *ICC* = 0.85), excellent internal consistency[48, 49]
SS average stepping activity^b^	Wearable sensors (Actigraph)	Actigraph GT3X + *(Actigraph Pensacola, FL, USA)* triaxial accelerometer; raw acceleration data are converted into activity counts per minute	Step activity monitors have been shown to have excellent test–retest reliability on 3-day monitoring (*ICC* > 0.9); criterion validity published in stroke [50–53]
CP/SS autonomy support environment	Family Care Climate Questionnaire FCCQ-CP/FCCQ-SS	14-item, Likert-type scale. Higher scores/higher autonomy support perception	Internal consistency > 0. 0; construct validity supported—higher FCCQ-SS scores related to SS lower perception of criticism, higher family emotional involvement—higher satisfaction with family support (*p* ≤ .05) [54]
CP strain	Caregiver Strain Index—CSI (modified)	Thirteen-item questionnaire, binary yes/no; higher score/higher strain	Good reproducibility and validity in stroke carepartners, Cronbach’s alpha of 0.83 [55–58]
CP family conflict	Family Caregiver Conflict Scale (FCCS) about Stroke Recovery	15-item, Likert-type scale higher scores/higher conflict	Established content/construct validity in stroke CP; reliability Cronbach’s alpha of 0.93 [59]
CP well-being related to caregiving	Bakas Caregiving Outcome Scale (BCOS)	Fifteen items; 7-point scale; higher scores/more positive caregiving outcomes	Satisfactory reliability and validity in stroke carepartner, Cronbach’s alpha of 0.90 [60]
**CARE-CITE-Gait usability**			
CP experience in CARE-CITE	Exit interview questionnaire	Three sections assessing confidence in care, value of participation, and aspects of CARE-CITe	Interview guide will be reviewed by content and qualitative experts prior to use
CP satisfaction with CARE-CITE	Feedback forms at end of CARE-CITE modules	5-item, Likert-type scale; higher scores/higher satisfaction	Interview guide will be revised by content and qualitative experts prior to use
CP experience using CARE-CITE	Post-Study System Usability Questionnaire (PSSUQ)	19-item, Likert-type scale. Lower scores/greater usability of instrument	Established reliability and validity [31]

*SS* Stroke survivor, *CP* Carepartner, CCG-CARE-CITE-Gait, *BP* blood pressure, *HR* Heart rate, *O*_*2*_ Oxygen saturation, *5STS* 5-times sit to stand, *TUG* Timed up and go test, *SIS* Stroke impact scale, *FCCQ-SS* Family Care Climate Questionnaire for stroke survivor, *ABC* Activities-specific Balance Confidence Scale, *CSI* Caregiver Strain Index, *BCOS* Bakas Caregiving Outcomes Scale, *FCCS* Family Caregiver Conflict around Stroke Recovery Scale, *FCCQ-CP* Family Care Climate Questionnaire for carepartner, *PSSUQ* Post-Study System Usability Questionnaire PC.

^a^Additional administration during home visit week 1 and week 4.

^b^Accelerometers worn at home for 7 days

#### Additional assessments

Stroke survivor medical records and dyad information questionnaires will be used to document data about participant demographics (age, gender), marital status, education level, income, work status, self-identified race and ethnicity, comorbidities, COVID (past history of testing positive and vaccinations), and current medications. Using the dyad zip code, we will determine the Area Deprivation Index (ADI) [33], which characterizes the relative disadvantage of an individual or social network using several US Ccensus indicators of employment, housing, poverty, and education [34]. Taken together, this collection of data will guide recruitment to ensure a diverse and inclusive participant sample.

#### Step activity data

To gain information about real-world stepping activity at home and in the community, the stroke survivor will be provided with a step activity monitor (Actigraph GT3X + (Pensacola, FL, USA)), to be worn on the paretic and non-paretic ankles for 7 days in order to obtain at least 3 days of step activity data. Participants will be provided with verbal and written instructions to don the device upon waking and doff prior to sleeping, removing the device during the day only for bathing, or taking part in water-based activities. Daily stepping activity for the paretic and non-paretic leg before and after the intervention period will be calculated.

#### Data management and analysis

REDCap (Research Electronic Data Capture) [35] electronic database will be used to store the quantitative data. REDCAP is a secure (compliant with United States healthcare confidentiality legislation requirements), web-based application designed to support data capture for research studies, providing (1) an intuitive interface for validated data entry, (2) audit trails for tracking data manipulation and export procedures, (3) automated export procedures for seamless data downloads to common statistical packages, and (4) procedures for importing data from external sources. The study coordinator will be trained in REDCap processes and will complete all data entry. Data will be double-checked for verification by a second REDCap-trained study staff. REDCap creates a comma-separated value file and SAS program to create an analytic dataset. These files and all resulting datasets, programs, and results are stored on a HIPAA-compliant Emory University Rollins School of Public Health server.

Standard data cleaning, identification of missing data, and internal consistency reliability for standardized scales will be completed. We will investigate whether missing data is related to treatment and baseline factors but do not anticipate missing data being a limitation if lost-to-follow-up rates in this new clinical trial are similar to other stroke studies performed in the PI (SB) and Co-I (TK) labs. Primary quantitative statistical analyses will be performed by the study statistician (GC) using SAS statistics for Windows (Version 9.4), and accelerometry data analysis (stroke survivor stepping activity) will be performed using ActiLife software (Version 6.13.4). Descriptive statistics (e.g., frequencies, means, ranges, standard deviations) will be calculated for all relevant variables, as well as to identify unusual or suspect values requiring review and confirmation. Additional descriptive statistics will be calculated for feasibility measures of recruitment rate, retention, and intervention adherence (described under the “Feasibility” section). To determine estimates of variability (for postulating effect sizes to design next phase clinical trials), the mean and median changes from baseline to 1 month and standard errors of the means will be calculated. To provide preliminary information about possible feasibility study intervention effects, confidence intervals for the difference in mean changes for major study variables will be reported as descriptive statistics. Graphical methods will be used to investigate outliers and investigate the consistency of the changes over time. Estimates of intercorrelations among the study variables will be calculated to gain insight into potential variables that could influence response to the intervention and guide the design of future studies. To understand potential relationships and possible confounding factors, we will examine stroke survivor comorbidities and the biological variables of carepartner age, sex, gender, and relationship of carepartner to stroke survivor. The second phase of the analysis will include one-way repeated measures analysis of variance (ANOVA) to evaluate differences between baseline, post, and follow-up timepoints. Tukey’s pairwise comparisons will be used to determine which time points are different if necessary. Level of significance of 0.05 will be used. No adjustments will be used to control for multiple study variables.

#### Safety

While no significant risks have been associated with home-based gait and mobility training, inherent risks associated with the intervention involve fatigue, muscle soreness, and falls. To minimize these risks, additional content related to mobility safety has been incorporated into the CARE-CITE-Gait modules. Safety issues will be discussed during home visits with support for collaborative problem-solving around maximizing safety during mobility training, and gait belts will be used to reduce fall risks during evaluations and as appropriate during home visits. Additionally, stroke survivor vital signs will be monitored during all study assessments and visits. The interventionist will monitor adverse events throughout the intervention period with frequent assessments of pain, fatigue, and falls as well as any medical appointments that may reveal changes in medications or medical conditions. Adverse events will be documented and categorized based on the Emory University Internal Review Board Protocol. The PI will be notified to evaluate and determine if an event is serious or related to the intervention and, in collaboration with the study team, will determine whether safety concerns warrant termination of participation in the study. Safety considerations arising throughout the study will be discussed regularly during study meetings. Though risks associated with carepartner participation in the review of the CARE-CITE-Gait modules are minimal, psychosocial assessments evaluating quality of life and mood will be monitored. In the case of any areas of concern (e.g., potential depressive symptoms), the PI will recommend a referral to their primary healthcare provider for further assessment. Due to the minimal study participation risks, a Data Safety and Monitoring Board was not established.

#### Auditing

All study records will be available for review by authorized representatives of the Foundation for Physical Therapy Research, regulatory agencies, and the Emory University Institutional Review Board to monitor study safety, progress, and procedures for quality assurance.

#### Dissemination

Dissemination of study results to academic communities will occur through peer-reviewed manuscripts and regional and national conference presentations. The study team will work collaboratively with the funding agency to share results based on agency requirements. The PI will meet regularly with the study team to revise the proposed dissemination plan and discuss authorship guidelines. For nonacademic communities, the PI will schedule presentations with community stroke groups and therapy clinics involved in study recruitment. A lay summary of study findings will be created for distribution based on participant requests.

## Discussion

This protocol describes the methodology to evaluate the feasibility of a Carepartner-Integrated Telehealth Gait Rehabilitation Program during stroke recovery. The study findings will provide valuable insights regarding innovative home-based rehabilitation that engages carepartners and support a future study testing the efficacy of CARE-CITE-Gait intervention. Web-based approaches like CARE-CITE-Gait increase accessibility to content outside of traditional clinic hours and reduce transportation-related barriers. While many carepartners may still lack resources and skills to use web-based approaches, the pandemic has rapidly increased the adoption of these technologies, and a recent study [36] found that 86.1% of stroke survivors and carepartners had Internet access. Implementing telerehabilitation delivery of carepartner interventions offers promising and scalable alternatives to improve access to care. Results will provide important preliminary estimates of efficacy and components of variability as well as inform future randomized clinical trial sample size power calculations. Data gathered regarding recruitment, retention, adherence rates, adverse events, outcome measure appropriateness, and participant exit interviews will inform the feasibility and justification of a definitive trial of CARE-CITE-Gait. Our proposal will lay the foundations for several research trajectories with the long-term goal of developing more personalized, precise, and efficacious family-focused rehabilitation interventions.

## Data Availability

The datasets generated and/or analyzed during the current study are not publicly available due to some data containing information that could compromise research participant privacy/consent but are available from the corresponding author [sb] on reasonable request.

## Abbreviations

CARE-CITE-Gait: Carepartner and Collaborative Integrated Therapy-Gait
FCCS: Family Caregiver Conflict Scale about Stroke Recovery
FCCQ: Family Care Climate Questionnaire
CSI: Caregiver Strain Index
BCOS: Bakas Caregiving Outcomes Scale
SIS: Stroke Impact Scale
5STS: 5-Times sit to stand
TUG: Timed up and go test
SIS: Stroke Impact Scale
ABC: Activities-specific Balance Confidence Scale
PI: Principal investigator
Co-I: Co-investigator
CP: Carepartner
SS: Stroke survivor
